# Emission Characteristics of InGaN/GaN Core-Shell Nanorods Embedded in a 3D Light-Emitting Diode

**DOI:** 10.1186/s11671-016-1441-6

**Published:** 2016-04-22

**Authors:** Byung Oh Jung, Si-Young Bae, Seunga Lee, Sang Yun Kim, Jeong Yong Lee, Yoshio Honda, Hiroshi Amano

**Affiliations:** Department of Electrical Engineering and Computer Science, Nagoya University, Nagoya, Aichi 464-8603 Japan; Akasaki Research Center (ARC), Nagoya University, Nagoya, Aichi 464-8603 Japan; Center for Integrated Research of Future Electronics (CIRFE), Institute of Materials and Systems for Sustainability (IMaSS), Nagoya University, Nagoya, Aichi 464-8603 Japan; Center for Nanomaterials and Chemical Reactions, Institute for Basic Science (IBS), Daejeon, 305-701 Korea; Department of Materials Science and Engineering, Korea Advanced Institute of Science and Technology (KAIST), Daejeon, 305-701 Korea

**Keywords:** Gallium nitride, Nanorod, Core-shell structure, Light-emitting diodes

## Abstract

We report the selective-area growth of a gallium nitride (GaN)-nanorod-based InGaN/GaN multiple-quantum-well (MQW) core-shell structure embedded in a three-dimensional (3D) light-emitting diode (LED) grown by metalorganic chemical vapor deposition (MOCVD) and its optical analysis. High-resolution transmission electron microscopy (HR-TEM) observation revealed the high quality of the GaN nanorods and the position dependence of the structural properties of the InGaN/GaN MQWs on multiple facets. The excitation and temperature dependences of photoluminescence (PL) revealed the *m*-plane emission behaviors of the InGaN/GaN core-shell nanorods. The electroluminescence (EL) of the InGaN/GaN core-shell-nanorod-embedded 3D LED changed color from green to blue with increasing injection current. This phenomenon was mainly due to the energy gradient and deep localization of the indium in the selectively grown InGaN/GaN core-shell MQWs on the 3D architecture.

## Background

III-nitride-based materials are a promising source of energy-saving solid-state lighting, particularly for light-emitting diodes (LEDs). There has been significant technological development in the field of nitride-based solid-state lighting over the past few decades since the discovery of the low-temperature buffer growth technique and a method of magnesium activation [1, 2]. Although considerable improvements in the performance of LED devices have been demonstrated, *c*-plane polar gallium nitride (GaN)-based LEDs still have unsolved problems that adversely affect their performance, which are caused by piezoelectric and spontaneous polarization [3, 4]. To solve such problems, different crystal planes, particularly nonpolar facets such as the *m*- and *a*-planes, can be applied to an InGaN/GaN multiple-quantum-well (MQW) heteroepitaxial structure as basal facets [3]. LED structures based on nonpolar facets are expected to avoid piezoelectric-field-related issues such as efficiency droop. However, the use of an *m*-plane bulk substrate increases the cost of devices. Moreover, a nonpolar-plane-based heteroepilayer grown on *a*-plane GaN or *r*-plane sapphire substrates is still needed to prevent the formation of structural defects such as prismatic and basal stacking faults as well as partial dislocations to ensure sufficient crystal quality [5, 6]. In fact, the results of research on GaN nanorods (NRs) and nanowires (NWs) have produced advances and interesting outcomes in a number of application fields [7–9]. Among them, the selective-area growth (SAG) of GaN NRs is a particularly promising alternative for producing nonpolar-based LED structures without increasing the production cost of devices or introducing structural defects owing to their unique characteristics. Motivated by these advantages, there has been tremendous effort to realize high-efficiency three-dimensional (3D) LEDs; indeed, 3D LEDs with full-color (or white light) emission have been demonstrated by geometrically emissive color mixing or phosphor-based wavelength conversion [7, 10–12]. Furthermore, owing to the recent progress in an epilayer transfer technique, the fabrication of flexible displays through the solid-state 3D LEDs is now feasible [13, 14]. However, NR-based 3D LEDs inevitably include several crystal facets, in contrast to a planar epilayer. This strongly affects the light emission properties of core-shell MQWs such as spectrum broadening and indium localization, thereby making it necessary to study their optical properties in more detail [15, 16]. Therefore, in this study, we report the emission characteristics of InGaN/GaN core-shell NRs embedded in a 3D LED structure. To clarify the underlying physics of this core-shell structure, we investigated its structural properties, particularly the well and barrier thicknesses of the MQWs, by Cs-corrected scanning transmission electron microscopy (STEM). We also performed measurements of the temperature (*T*) and excitation power (*P*) dependences of the photoluminescence (PL) to reveal their optical properties by analyzing the behaviors of the emission energy, intensity, and linewidth. Additionally, the light emission properties of the core-shell-NR-embedded 3D LEDs are presented in detail.

## Methods

### Preparation of Pattern

A 3-μm-thick GaN epilayer with a (0002) preferential orientation doped with Si was utilized as a basal template for the SAG of GaN NRs by metalorganic chemical vapor deposition (MOCVD). RF magnetron sputtering was used to deposit a 30-nm-thick dielectric SiO_2_ growth-masking layer. After SiO_2_ deposition, nanoscale resin patterns with a hole-shaped array (aperture size, 190 nm; center-to-center distance, 920 nm) were produced by thermal nanoimprinting procedure. Then, CF_4_-based reactive ion etching (RIE) was performed to open the SiO_2_ selectively. Subsequently, the patterned template was cleaned with an organic solvent (acetone) for 10 min to remove polymer-based residues from the surface.

### Synthetic Process and 3D LED Fabrication

For the growth of the GaN NR array and subsequent core-shell layers, the patterned GaN template on sapphire substrates was loaded into a showerhead-type MOCVD chamber. Trimethylgallium (TMGa) and ammonia (NH_3_) were used as the precursors with flow rates of 15 sccm (78 μmol/min) and 5 slm (223.21 mmol/min), respectively. At the same time, tetramethylsilane (Si(CH_3_)_4_) was used as the n-type doping source with a flow rate of 5 sccm (0.0105 μmol/min). The synthesis of the well-defined GaN NR array was carried out in accordance with Hersee’s original pulsed-mode growth procedure with our optimal growth conditions under an ambient of pure hydrogen (H_2_) as the carrier gas. Details of this pulsed-mode growth process have been reported elsewhere [17, 18]. Subsequently, three pairs of InGaN/GaN MQWs were grown on the GaN NR array in the conventional growth mode at temperatures from 760 to 790 °C depending on the experiment. For MQW growth, trimethylindium (TMI) and triethylgallium (TEG) were used as reactants with nitrogen (N_2_) as the carrier gas. The reactant flow rates of TMI and TEG were fixed at 400 sccm (40.3 μmol/min) and 100 sccm (29.92 μmol/min), respectively, with a chamber pressure of 200 Torr. Finally, p-type GaN was grown with bis-ethylcyclopentadienyl magnesium (EtCp_2_Mg) as a precursor at a flow rate in the range of 150–250 sccm (0.24–0.4 μmol/min) for 90 min to ensure full coalescence. To form ohmic contacts, Ni/Au and Ti/Au metal bilayers were deposited by electron beam evaporation onto the p-GaN top surface layer and the n-GaN underlayer, respectively. To decrease the resistance between the metal and the semiconductor (GaN), the metal contacts were treated by rapid thermal annealing (RTA) at 550 and 300 °C after the deposition of the Ti/Au and Ni/Au metal bilayers, respectively.

### Characterization

Morphological and microstructural characterization was carried out by field-emission scanning electron microscopy (FE-SEM; Hitachi S-5200) and Cs-corrected STEM (JEM-ARM200F). The synthesized samples were milled using a dual-beam focused ion beam (DB-FIB; NOVA200) operating in the range of 5–30 kV before TEM investigation to obtain high-resolution (HR), bright-field (BF), and electron diffraction pattern (DP) images. To investigate the emission properties of the InGaN/GaN core-shell NR array, the temperature dependence of the PL was measured as a function of temperature from 12 to 300 K in a closed-cycle helium cryostat. The excitation power dependence of the PL was measured in the power density range of 7.6–764 W/cm^2^. For all the PL measurements, a 325-nm continuous-wave He-Cd laser (20 mW) was used as the excitation source, and the objective lens (×5) of a reflecting microscope was used to control the beam spot size (~20 μm) and simultaneously collect the signal. In this paper, we define the maximum excitation power density (=764 W/cm^2^) as 100 %. Moreover, the characterization of the electrical properties (*I*-*V* curve) and electroluminescence (EL) spectra of the 3D LED was performed simultaneously.

## Results and Discussion

**Fig. 1:**
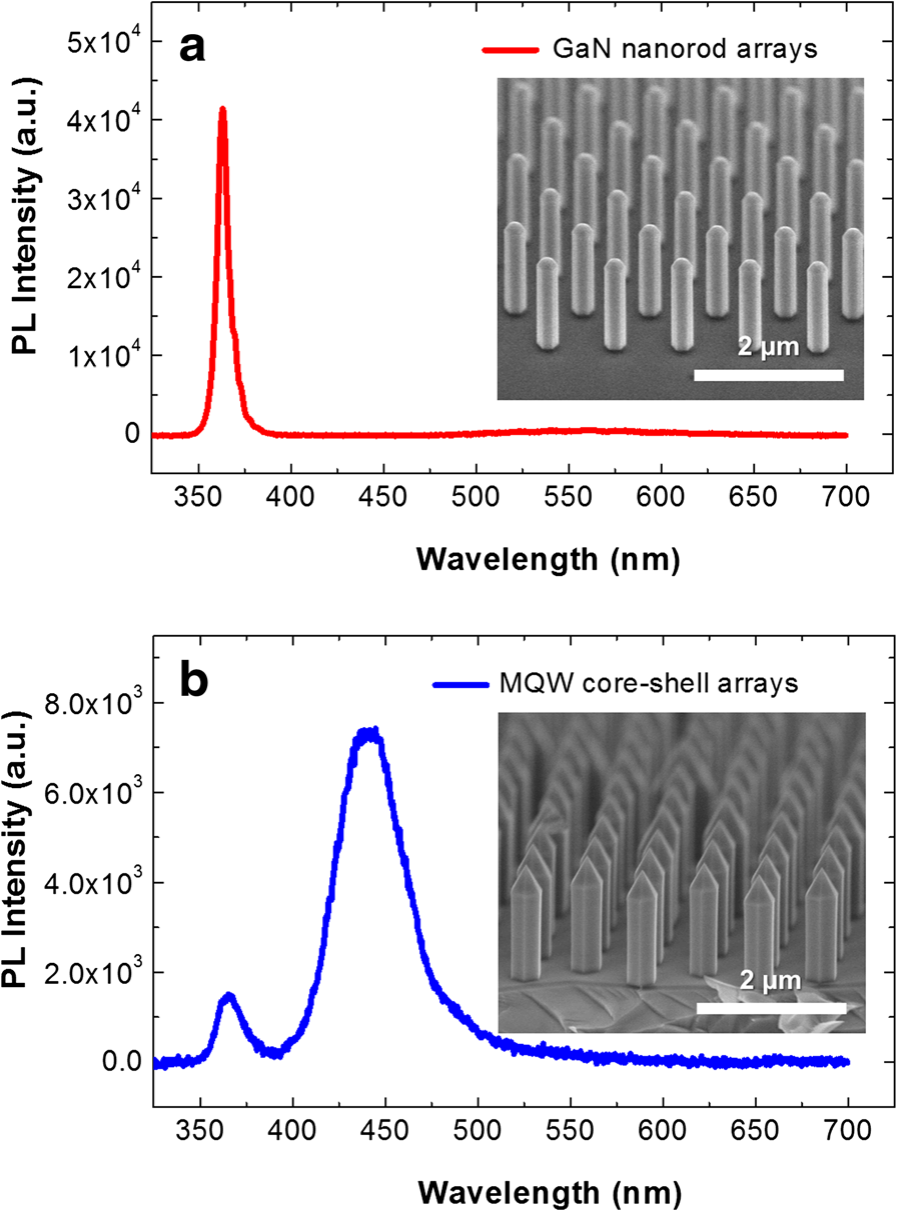
**a** Room-temperature PL spectrum and SEM image (*inset*) of a selective-area-grown GaN NR array. **b** Room-temperature PL spectrum and SEM image (*inset*) of the core-shell NR array including three pairs of InGaN/GaN MQWs

Figure 1 shows the room-temperature PL spectra of a selective-area-grown GaN NR array with three pairs of InGaN/GaN MQWs, and each inset FE-SEM image indicates the surface morphology. The PL spectrum of the GaN NR array exhibits typical behaviors of the high-quality GaN. A strongly dominant ultraviolet (UV) emission peak located at approximately 365 nm was observed, as shown in Fig. 1a. This intense UV peak is closely related to the recombination of free excitons, i.e., the near-band-edge (NBE) emission of the GaN crystal [1]. Furthermore, we observed hardly any defect-related yellow luminescence (YL) at approximately 550-nm wavelength, indicating the high optical and crystal quality of the GaN NR array. After the heteroepitaxial growth of the three pairs of InGaN/GaN MQWs, the structural morphology of the coaxial NR array still maintained its verticality, selectivity, and uniformity, as shown in the inset of Fig. 1b. The MQW light emission peak at approximately 445 nm dominated the optical characteristics of the PL spectrum in Fig. 1b. As before, hardly any YL-related emission was generated, indicating the high optical quality of the InGaN/GaN core-shell NR array. The PL spectrum of the NR array in this study showed similar emission properties to the previously reported cathodoluminescence (CL) spectrum of a single NR [17]. Hence, we expect that the light emission of the NR array will mainly originate from the MQWs on nonpolar $\left\{1\overline{1}00\right\}$*m*-plane sidewalls. A detailed structural analysis based on our TEM investigation is given later.

**Fig. 2:**
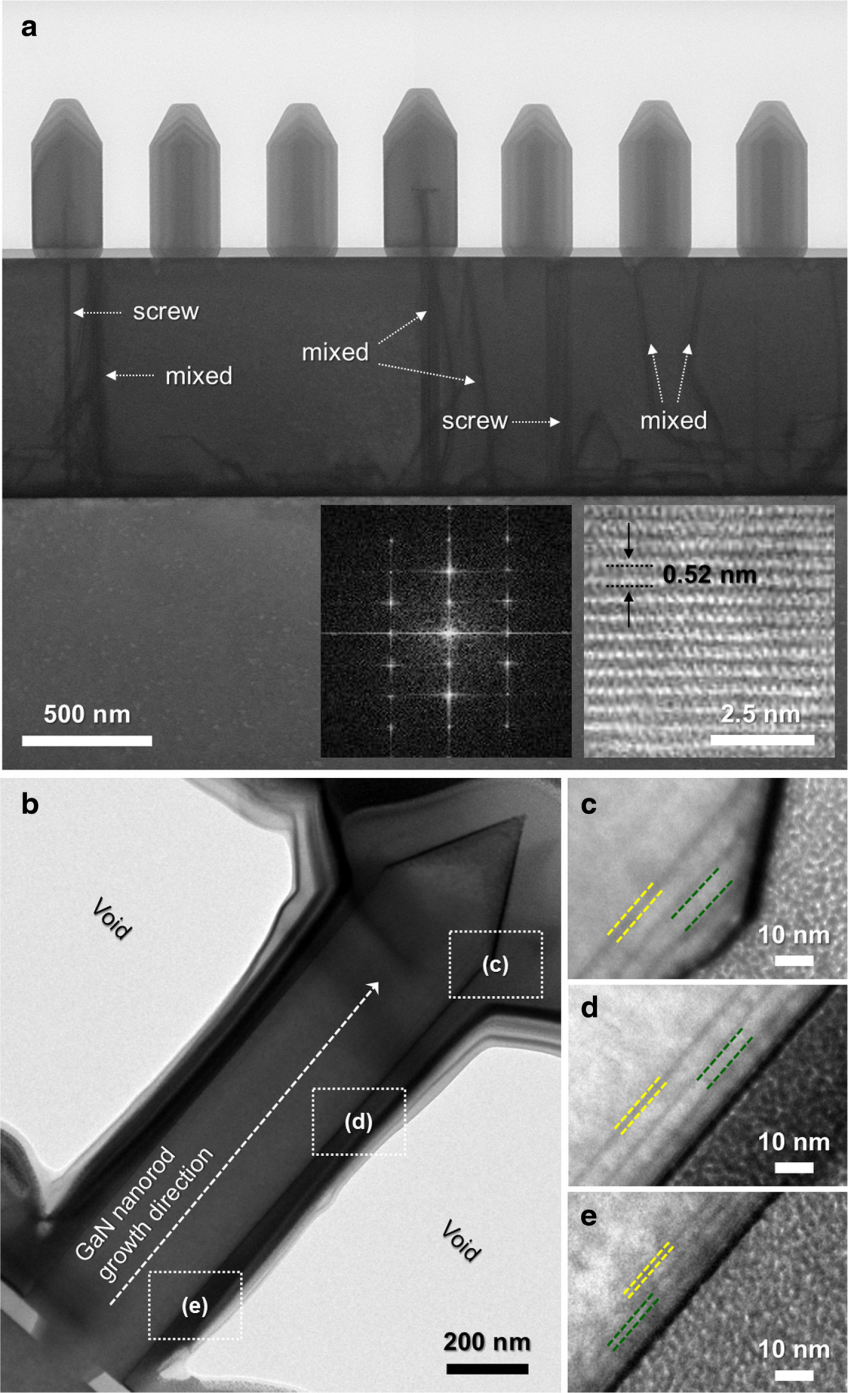
**a** Cross-sectional STEM image of GaN NRs showing the filtering of dislocations, suppressing their penetration into the GaN NRs. The HR image indicates the single-crystalline property of a GaN NR. The Fourier-filtered transformed (FFT) diffraction pattern (DP) was obtained from the HR image of a GaN NR. **b** Cross-sectional STEM image of the InGaN/GaN-MQW-based single core-shell NR under low magnification. The *squares* display the regions from which enlarged STEM images were obtained. **c**–**e** Thicknesses of QWs and QBs at different positions of the core-shell NR. The *yellow and green lines* indicate the QW and QB thicknesses, respectively

Figure 2a shows various outcomes of the cross-sectional TEM measurement such as HR, low-magnification BF, and DP images of the GaN NR array. The low-magnification cross-sectional STEM image shows selective-area-grown n-GaN NRs on a thick GaN underlayer captured in the $\left[11\overline{2}0\right]$ zone axis. Notably, there were a few dislocations caused by the lattice mismatch between the GaN underlayer and the sapphire substrate such as screw and mixed dislocations. Nevertheless, we clearly observed that most dislocations were filtered by the SiO_2_ mask and very few dislocations penetrated the inner core of the n-GaN NRs. This phenomenon leading to defect-free NRs can be described by the filtered-dislocation-effect model of nanoscale epitaxy [19]. We also confirmed the single-crystalline wurtzite properties of the n-GaN NRs by DP and HR analyses, as shown in the inset of Fig. 2a. Consequentially, we grew dislocation-free and highly crystalline GaN NRs. The low-magnification STEM image in Fig. 2b shows that the InGaN/GaN MQWs were uniformly coated on nonpolar $\left\{1\overline{1}00\right\}$*m*-plane sidewalls on the outside of the n-GaN NRs. Figure 2c–e shows the high-magnification STEM images used to examine the features of the MQWs grown on n-GaN NRs, where we mainly focused on the structural properties of the MQWs on the nonpolar $\left\{1\overline{1}00\right\}$*m*-plane sidewalls. The MQWs on the $\left\{1\overline{1}00\right\}$*m*-plane sidewalls on the outside of the n-GaN NRs were clearly stacked into dark (InGaN quantum well, QW) and bright (GaN quantum barrier, QB) layers, as shown in Fig. 2c–e. Generally, the MQW structures on the outside of the n-GaN NRs had a reasonably consistent multiple-stack layer; however, the thickness of the QW and QB layers varied according to the position of the core-shell NR. At upper positions, the MQWs were thicker than those at lower positions even in the same crystal facet. In other words, the thickness of the MQWs tended to increase from the bottom to the top of the core-shell NRs. In particular, the MQWs were thickest in the edge region at the interface between the $\left\{1\overline{1}00\right\}$ and $\left\{1\overline{1}01\right\}$ facets. This phenomenon may be related to the surface diffusion process in combination with the mean lifetime of the group III adatoms [20]. From the structural observation of other planes reported elsewhere, the undistinguishable thickness of semipolar QWs and the small area of indium-localized polar QWs have already been demonstrated [17]. Indeed, under the PL excitation, the *m*-plane surfaces mainly contributed to the obtained spectra, as shown in Fig. 1b, owing to their larger area than the other parasitic MQW planes. In most cases, these selectively grown structures had an indium composition gradient on the surface, thereby resulting in a significant *energy gradient* on the MQW surfaces accompanying the variation of the MQW thickness [17, 20, 21]. We hereafter use the term energy gradient to simultaneously reflect the indium composition gradient and the variation of the MQW thickness. Thus, it is necessary to study the optical properties of the core-shell NRs including the effect of the energy gradient. This will be discussed in detail in the next section on the basis of PL analysis.

**Fig. 3:**
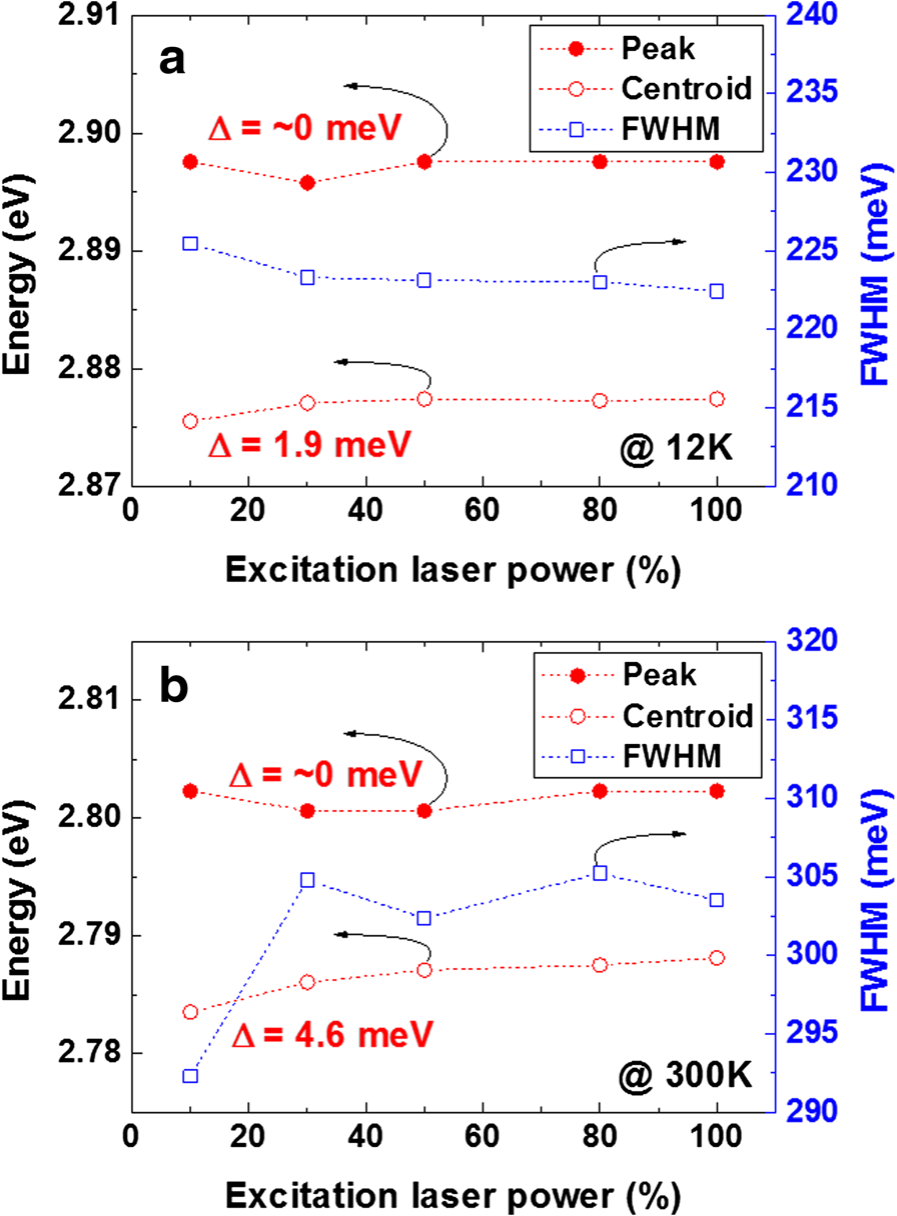
Excitation laser power dependences of PL emission peak energy, centroid peak energy, and linewidth (FWHM) at **a** 12 K and **b** 300 K

Figure 3 shows the peak energy, centroid peak energy, and full width at half maximum (FWHM) plotted as functions of the percentage excitation laser power (10–100 %) at low and high temperatures, i.e., 12 K (Fig. 3a) and 300 K (Fig. 3b). Note that we used the centroid peak energy to reflect the net shift of the broad PL, as is evident from the energy gradient of the *m*-plane MQWs. With increasing excitation laser power, almost no peak energy shift was observed at both low and high temperatures, showing the absolute reduction of the quantum-confined Stark effect (QCSE), which is related to the internal electric field (IEF), as a result of local epitaxy [22, 23]. The centroid peak energy exhibited blueshifts of only 1.9 and 4.6 meV at 12 and 300 K, respectively. At the same time, a slight decrease in the linewidth (the same as the FWHM in this communication) of 3 meV was observed at 12 K. Generally, there are two possible mechanisms explaining the dependence of the blueshift behavior on the PL excitation power density: one is Coulomb screening of the QCSE and the other is the band filling of localized states [24, 25]. The former usually produces linewidth narrowing, whereas the latter produces linewidth broadening [24, 26]. In our case, it is speculated that the band filling of localized states became the dominant process of the NR-based QWs with increasing excitation power at 12 K since the IEF almost disappeared. However, unlike the conventional broadening behavior of localized states, the linewidth of the NR-based QWs became narrow. Hence, we believe that the small decrease in the linewidth can be attributed to the energy gradient, thereby screening the energy transition of strongly localized states due to the simultaneous filling of carriers. On the other hand, we observed a large increase in the linewidth when the excitation power was increased from 10 to 25 % at 300 K, above which the linewidth remained almost constant (Fig. 3b). The initial peak broadening can be attributed to the dominance of thermally activated nonradiative recombination centers at the high temperature since they enable excited carriers to be readily recombined at high-energy extended states before reaching low-energy localized states [25]. With further increasing excitation power, radiative recombination became dominant after the rapid saturation of nonradiative centers, but the linewidth remained almost constant with a slight increase in the centroid peak energy. Thus, similar screening of the band-filling effect observed at 12 K also appeared at 300 K, as the constant linewidth is shown in Fig. 3b.

**Fig. 4:**
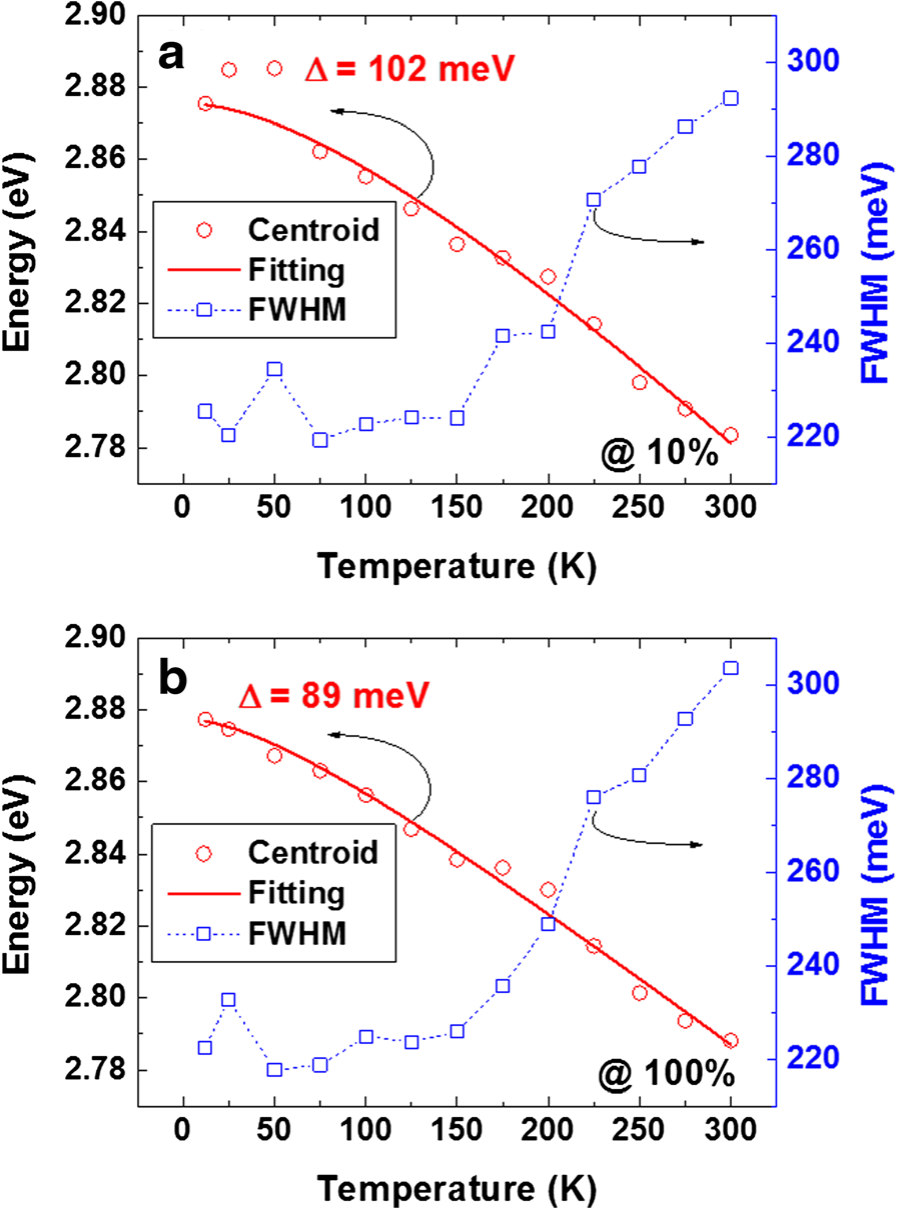
Temperature dependences of PL emission centroid peak energy and linewidth (FWHM) for excitation powers of **a** 10 % and **b** 100 %. The *red solid lines* represent the curves fitted using Varshni’s model

Figure 4 plots the temperature dependences of the centroid peak energy and FWHM for low and high excitation power densities, i.e., 10 % (Fig. 4a) and 100 % (Fig. 4b). For the excitation power density of 10 %, the centroid peak first undergoes a blueshift up to a temperature of ~50 K, above which it undergoes a continuous redshift, while a bending curve was observed at the full-delocalization temperature of ~175 K. Note that *c*-plane InGaN QWs typically exhibit an S-shaped peak shift (decrease-increase-decrease) with a corresponding W-shaped curve (decrease-increase-decrease-increase) for the FWHM [25, 26]. Regardless of the significant difference in the peak shift behavior between typical *c*-plane InGaN QWs and NR-based QWs, the linewidth of the NR-based QWs showed a similar temperature dependence to that of *c*-plane InGaN QWs. The linewidth slightly decreased up to a temperature of 25 K, and then increased up to a temperature of 50 K, which was similar to the temperature of the highest centroid peak energy. Furthermore, the linewidth decreased up to a temperature of 75 K and then steadily increased up to 300 K. Hence, it clearly showed a W-shaped temperature dependence. To explain in detail the different temperature dependences of the peak shift for conventional *c*-plane InGaN QWs and NR-based QWs, it is necessary to understand the possible carrier transfer mechanisms. Note that the excitation carrier density of the PL system was sufficiently low to study the carrier transfer mechanism (<1000 W/cm^2^). In general, at low temperatures from 12 to 50 K, the carrier transfer mechanism has been considered to be the carrier-hopping-dominant mechanism, indicating that weakly localized carriers can become thermally activated and relax into other strongly localized states caused by inhomogeneous potential fluctuations [27]. The slight narrowing of the linewidth is key evidence of this redistribution of weakly localized carriers [26]. In the NR structures, however, an energy gradient was induced in the InGaN QWs. Hence, it is speculated that the initial blueshift of as large as 10 meV for the NR-based QWs in the temperature range of 12–50 K was due to the anti-Stokes luminescence generated by the two-step absorption process, i.e., photon recycling, since there are overlapping states between a weakly localized state at a low-energy level and a strongly localized state at a high-energy level [28, 29]. As the temperature increased from 50 K, a large redshift of the centroid peak energy was observed. In fact, a large blueshift has been observed for *c*-plane InGaN QWs in a similar temperature range (60–150 K) since thermally enabled carriers can occupy the higher-energy levels of the localized states [25, 26]. Hence, the NR-based QWs should also follow this band-filling process in the same temperature range. However, in the NR-based QWs, multiple localized states can be generated owing to the energy gradient. As a consequence, the band-filling effect, i.e., the blueshift behavior, can be weakened. This is consistent with the slope of 0.35 meV/K observed in the temperature range of 75–150 K, compared with 0.45 meV/K in the temperature range of 200–300 K. In general, the redshift becomes dominant with increasing temperature owing to the temperature-dependent dilatation of the lattice and the electron-lattice interaction [30, 31]. This temperature-induced redshift of the peak energy can be described by Varshni’s empirical model *E*(*T*) = *E*(0) − *αT*^2^/(*T* + *β*), where *E*(0) is the energy gap at 0 K and *α* and *β* are fitting parameters [32]. The red solid lines in Fig. 4a are the fitting curves obtained using Varshni’s model with *E*(0) = 2.877 eV, *α* = 0.39 meV, and *β* = 89 K. The fitting curves closely match the experimental results except in the initial temperature range of 12–50 K. Meanwhile, the small increase and decrease results in the linewidth in the temperature range of 25–75 K can be assigned to the crossover from the nonthermalization to thermalization of localized excitons (increase) and to the further redistribution of mobile carriers (decrease) [33, 34]. The slow increase in the linewidth 5 meV in the temperature range of 75–150 K indicates that the change in the linewidth caused by carrier thermalization is screened by the overlapping of the localized states due to the energy gradient. At 175 K, a slow increase in the centroid energy and an abrupt increase in the linewidth were observed, indicating that the excited carriers had almost reached the free-exciton ground state. Then, the temperature-induced bandgap shrinkage became more dominant; thereby, a steeper slope of the peak energy shift and linewidth broadening was observed. The temperature dependence of the PL results at the higher excitation power density (100 %) is plotted in Fig. 4b. The main difference was found at low temperatures (<50 K), where a monotonic redshift of the centroid peak energy was observed. It is believed that a high excitation power density enables a sufficient number of injected carriers to occupy both weakly localized states and strongly localized states for the carrier-hopping effect to be reduced. Hence, a decrease in the linewidth was not observed at the same temperature as for the excitation power density of 10 %. Furthermore, the small peak in the linewidth was shifted from 50 to 25 K owing to the increase in the injected carrier density in the strongly localized states. Above 75 K, the redshift of the centroid peak energy and the linewidth broadening behavior were similar to those observed at 10 % PL excitation power density, where the same carrier relaxation mechanism is considered to have occurred. Note that the slope was found to be 0.33 eV/K at 75–150 K and 0.42 eV/K at 200–300 K, also indicating a small band-filling effect on the peak shift behavior. The curve fitted using Varshni’s model was also in good agreement with the experimental results, as shown by the red solid line in Fig. 4b. The total energy shift over the entire temperature range was slightly reduced from 102 to 89 meV upon increasing the PL excitation power density.

**Fig. 5:**
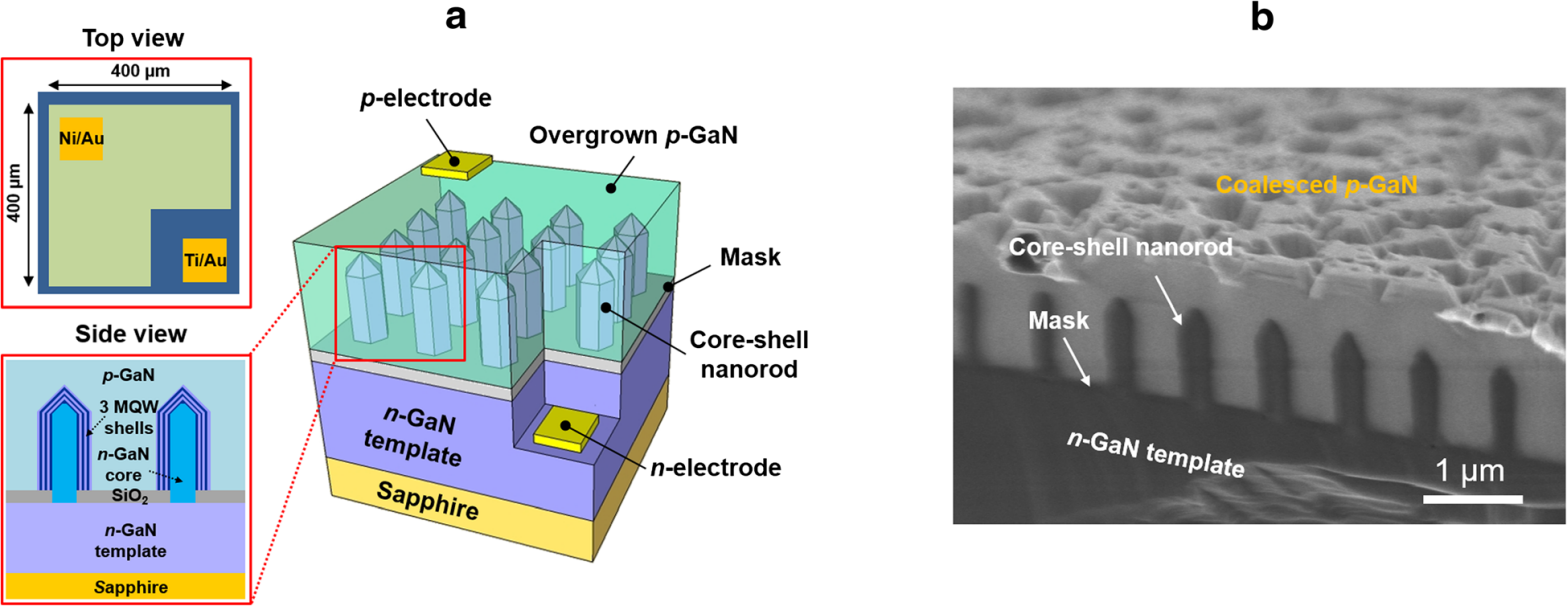
**a** Schematic of fabricated 3D LED device. The *upper and lower panels* on the *left side* show a top view and side view of the 3D LED, respectively. **b** SEM image of the 3D LED showing fully overgrown p-GaN on core-shell NRs

We fabricated a core-shell NR-array-embedded 3D LED to observe emission properties under electrical bias. A schematic of the fabricated device is shown in Fig. 5a. The upper and lower panels on the left show a top view and side view of the 3D LED, respectively. The mesa layer of 400-μm width was formed by dry etching. Note that we performed p-GaN overgrowth on InGaN/GaN core-shell NRs. The planarization of the p-GaN region enabled the use of a simple fabrication process compared with the fabrication by the direct contact of 3D structures. For example, we can avoid the issue of conformal covering of current-spreading layer, the filling of empty spaces for electrical passivation, and the low-doping issue of semi- and/or nonpolar planes [11–13, 35, 36]. Indeed, the electrical operation of an embedded-type 3D LED has been demonstrated elsewhere [7, 15, 37]. However, the coalesced p-GaN obtained under the present growth conditions still exhibited severe surface roughness, as shown in Fig. 5b. Hence, we intentionally reduced the contact resistivity of the p-electrode by using ohmic Ni/Au metal layers instead of a common current-spreading layer such as an indium tin oxide (ITO). Then, the light output power was collected on the bottom side (i.e., the sapphire side) because of the opaque p-electrode.

**Fig. 6:**
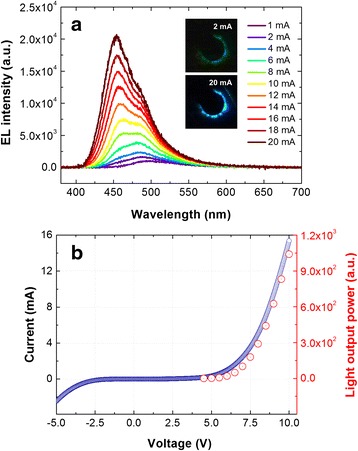
**a** EL spectra of the InGaN/GaN core-shell NR-embedded 3D LED obtained at various injection currents from 1 to 20 mA. The *insets* are photographs of the light emission. **b** *I*-*V*-*L* characteristics of the core-shell NR-embedded 3D LED

The operation properties of this 3D LED were investigated by EL and current-voltage-light output power (*I*-*V*-*L*) characterization. Figure 6a shows the EL spectra for various injection currents. The dominant EL emission wavelength was observed to be 500 nm at 1 mA, corresponding to the near-green region. However, the EL emission was shifted to the blue region with a slight shoulder peak when the injection current was increased to 20 mA, which is related to the dissimilar MQW layers on the different crystal facets of the GaN NR caused by the current injection path [7, 17]. The upper and lower insets of Fig. 6a are optical microscope images of the device light emission at the injection currents of 2 and 20 mA, respectively. For both injection currents, the EL was concentrated close to the contact, which may have been due to the high resistivity of the p-GaN. It is well known that the current spreading of LEDs is strongly related to the layer thickness as well as the resistivity [38]. We used a p-GaN layer with a high thickness of >1 μm, while the coalesced p-GaN layer included unexpected surface roughness, as shown in Fig. 5b. We believe that the unusual growth, i.e., coalescence from selectively grown structures, might have resulted in the higher resistivity of p-GaN than that of p-GaN obtained by conventional two-dimensional (2D) growth, thereby resulting in weak current spreading. We show the *I*-*V*-*L* characteristics of the core-shell NR-embedded 3D LED in Fig. 6b. The *I*-*V* curve showed the rectifying feature of the LED with a turn-on voltage of approximately 6 V. The current began to increase rapidly above the turn-on voltage, resulting in increased light output power. Also, the leakage current was observed to be approximately 2 mA at −5 V. Two possibilities may account for the leakage current: the unsuccessful growth of GaN NRs within the nanopattern and a current path through the semipolar plane [11]. Therefore, we expect that the leakage problem can be suppressed by employing a suitable patterning process or optimizing the growth conditions such as by increasing the aspect ratio of the core-shell NRs and the use of highly doped p-GaN with a smooth morphology.

**Fig. 7:**
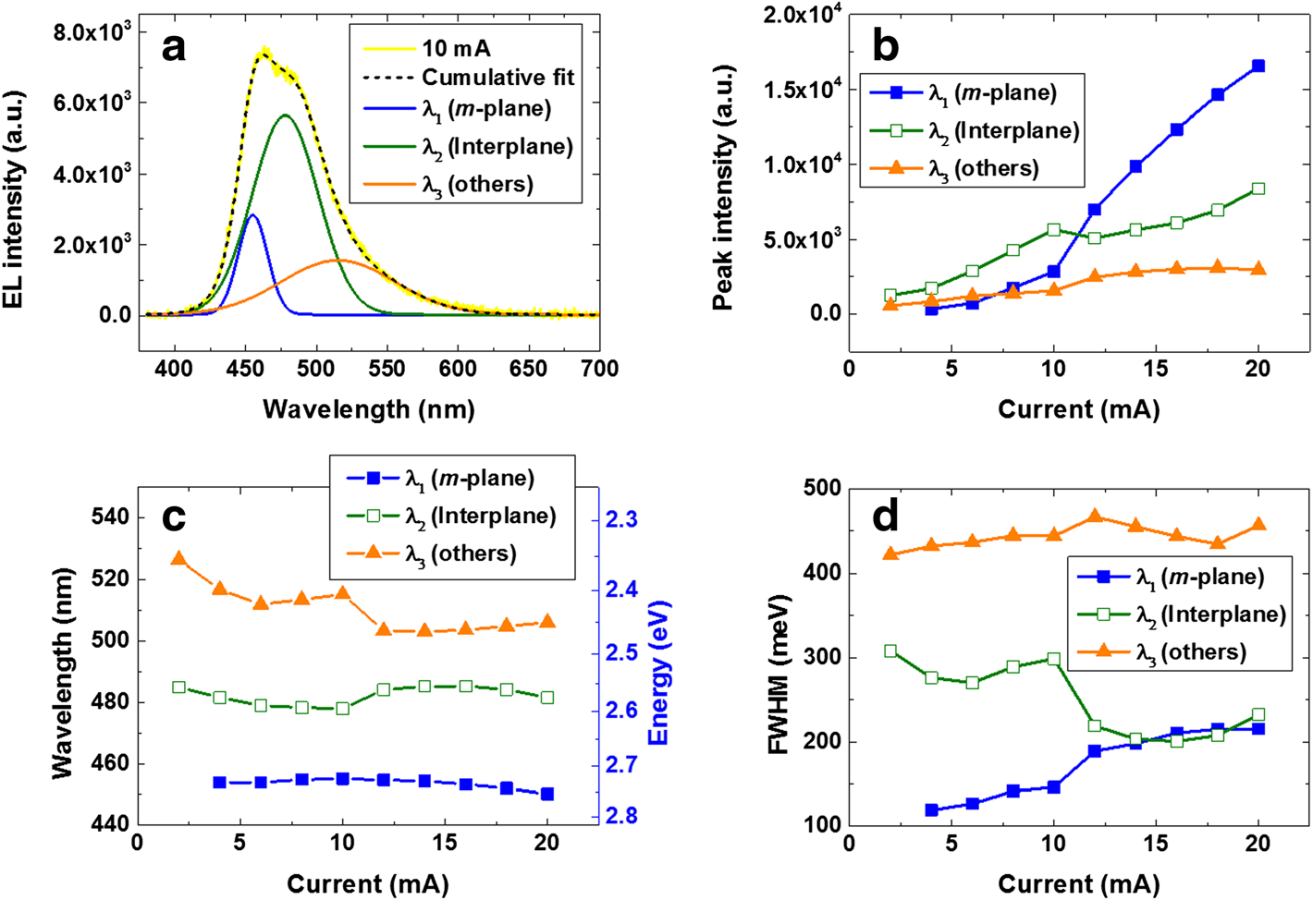
**a** Representative example of triple peak fitting for EL spectrum at 10 mA. Plots of **b** peak intensity, **c** peak wavelength with corresponding energy, and **d** FWHM of fitted spectra as a function of injection current. Note that *λ*_1_, *λ*_2_, and *λ*_3_ correspond to the *m*-plane, interplane, and other planes, respectively

For more detailed analysis of the EL, we performed multiple peak fitting of EL spectra shown in Fig. 6a. A representative example of triple peak fitting for the EL spectrum at 10 mA is presented in Fig. 7a. From the above discussion of the InGaN layers on the 3D structures, we assumed that three components mainly contribute to the EL, i.e., the *m*-plane (*λ*_1_), the interplane (*λ*_2_) between the sidewall and the pyramidal facets, and other planes (*λ*_3_) such as the *c*-plane and/or semipolar planes. On the basis of previous reports of indium incorporation rates, we assigned the order of the wavelengths of the fitted peaks in the spectral range to be *λ*_1_ < *λ*_2_ < *λ*_3_ [17, 39–41]. Indeed, the cumulative curve (dashed line) with the three fitted spectra closely matched the actual EL spectrum (yellow line), indicating the appropriate allocation of the fitting peaks. Figure 7b shows the peak intensity of the fitted spectra as a function of the injection current. Notably, the peak intensity of *λ*_2_ was highest under the low injection current (≤10 mA), while that of *λ*_1_ became dominant under the high injection current (>10 mA). This switching behavior of the EL peak intensity upon increasing the applied bias is the main difference from the results of the PL characterization. According to the analysis of the PL peak, the PL intensity was mainly affected by the area of excitation on the oriented surface, thereby resulting in the prominent emission of *m*-plane QWs in this study. On the other hand, EL emission was first observed from regions with a high level of indium incorporation since these regions became quickly the electrical turn-on state, and then, the recombination of regions with a low level of indium incorporation was enabled. To investigate the degree of indium incorporation in each component, plots of the EL peaks are presented in Fig. 7c. It can be seen that the peak of *λ*_1_ was located at an energy of >2.72 eV and was hardly shifted compared with other peaks. This was in good agreement with the peak positions and shifts of the excitation-power-dependent PL shown in Fig. 3. The peak positions of the second intense *λ*_2_ peak also coincided with the interplane indium localization, as was evident from HR-CL characterization reported elsewhere [17]. Although there is some deviation among the peak positions due to the use of a simple computational fitting method, it is speculated that the large peak shift of *λ*_3_ is related to the higher piezoelectric field of the *c*-plane or the semipolar planes compared with that of the *m*-plane emission. However, the PL emission corresponding to *λ*_3_ was indistinct due to its small area and weak intensity. Figure 7d shows plots of the FWHM of the fitted EL spectra. Although *λ*_3_ has the broadest FWHM, it did not make the main contribution to the total light emission, as discussed earlier. Interestingly, the prominent FWHM of ~300 meV for *λ*_2_ at the low injection current (≤10 mA) converged to the FWHM of ~200 meV as the injection current increases, which corresponded to the FWHM for *λ*_1_ at the high injection current (>10 mA). Note that the change in the FWHM was similar to that of the temperature-dependent PL spectra in Fig. 4. Hence, we consistently observed the deep localization behavior of the embedded 3D LED from the results of both PL and EL analyses.

## Conclusions

We have demonstrated and analyzed InGaN/GaN core-shell NRs embedded in a 3D LED. The high quality of the core GaN NRs was proved from the dislocation filtering of the structure and the prominent NBE PL emission. Owing to the vapor-phase diffusion and surface diffusion of the selectively grown 3D structures, the InGaN/GaN MQW core-shells that formed on the GaN NRs suffered from an indium compositional gradient and thickness variation, thereby resulting in an energy gradient. Under the excitation-power-dependent PL characterization, the emission on the *m*-plane surface mainly contributed to the total PL spectra, as observed from the suppressed peak shift. The temperature-dependent PL characterization revealed a monotonic peak shift and linewidth variation with increasing the temperature, indicating the strong indium localization of the *m*-plane QW layer with the energy gradient. Through the demonstration of an electrically driven 3D LED, the EL properties were also characterized. At a low injection current, the EL spectra were mainly affected by the regions of localized indium concentration such as at the top of *m*-plane MQWs. Upon increasing the injection current, the *m*-plane emission became more prominent and the peak shift was suppressed. Further EL peak analysis showed good agreement with the result of PL characterization in terms of the intensity, peak energy, and FWHM. Therefore, the results of this paper are considered to contribute to understanding of the emission properties of the 3D LEDs, thereby helping to achieve highly efficient devices through further control of the growth and fabrication.
